# UPLC-QTOF/MS^E^ and Bioassay Are Available Approaches for Identifying Quality Fluctuation of Xueshuantong Lyophilized Powder in Clinic

**DOI:** 10.3389/fphar.2018.00633

**Published:** 2018-06-15

**Authors:** Zhi-Rui Yang, Zi-Hao Wang, Jin-Fa Tang, Yan Yan, Shi-Jun Yue, Wu-Wen Feng, Zheng-Yuan Shi, Xin-Tong Meng, Cheng Peng, Chang-Yun Wang, Da-Li Meng, Dan Yan

**Affiliations:** ^1^Beijing Key Laboratory of Bio-characteristic Profiling for Evaluation of Rational Drug Use, Beijing Shijitan Hospital, Capital Medical University, Beijing, China; ^2^School of Traditional Chinese Materia Medica, Shenyang Pharmaceutical University, Shenyang, China; ^3^Department of Pharmacy, the First Affiliated Hospital of Henan University of Chinese Medicine, Zhengzhou, China; ^4^Department of Pharmacy, Chengdu University of Traditional Chinese Medicine, Chengdu, China; ^5^Key Laboratory of Marine Drugs (Ministry of Education of China), School of Medicine and Pharmacy, Ocean University of China, Qingdao, China

**Keywords:** Xueshuantong lyophilized powder, quality fluctuation, adverse drug reaction, UPLC-QTOF/MS^E^, bioassay

## Abstract

Xueshuantong Lyophilized Powder (XST), consisting of a series of saponins extracted from *Panax notoginseng*, is widely applied to treat acute cerebral infarction, stroke, and coronary heart disease in China. However, most adverse drug reactions (ADR) in clinic are caused by quality problems of XST. In this study, six batches of certainly abnormal, four batches of possibly abnormal XST, and eight batches of normal XST were obtained from the clinical practice. Their quality fluctuations were identified by ultra-performance liquid chromatography coupled with an electrospray ionization quadrupole time-of-flight mass spectrometry operating in MS^E^ mode (UPLC-QTOF/MS^E^) and bioassays including antithrombin and proplasmin assay. Fourteen potential components responsible for clinical ADR were identified by UPLC-QTOF/MS^E^, especially ginsenoside Rg1, Rg3, Rb1 and notoginsenoside R1. In addition, 83.3% (5/6) and 50.0% (3/6) certainly abnormal samples could be identified by UPLC-QTOF/MS^E^ and bioassay, respectively. Interestingly, further integration of the two methods could entirely identify all the certainly abnormal samples and inferred that all the possibly abnormal samples were closely related to their quality fluctuation. It indicates that it is advisable to combine UPLC-QTOF/MS^E^ and bioassay for identifying quality fluctuation of XST, and thus reduce its ADR in clinic.

## Introduction

Herbal injections are one kind of the modern preparations derived from traditional Chinese medicine. With their powerful and rapid therapeutic effects on specific diseases (e.g., cardiovascular disease, cancer), herbal injections are attracting more and more attention in mainland China, Hong Kong, and Taiwan (Normile, 2003; Luo et al., 2015). Currently, more than 130 types of herbal injections are applied clinically among about 400 million patients per year, generating approximately annual sales of four billion US dollars (Zhang et al., 2014). However, data from China Food and Drug Administration (CFDA) showed that 127 thousand cases of adverse drug reactions (ADR) were induced by herbal injections, and it is estimated to account for 53.7% of ADR induced by traditional Chinese medicine (China Food and Drug Administration, 2016). The Chinese Government is deeply concerned about the considerable number of ADR caused by the quality problem of herbal injections and has made an announcement to improve their quality standards (General Office of the State Council of the People's Republic of China, 2017; Xu et al., 2018). Therefore, it is important to develop the quality standards of herbal injections to ensure their clinical safety.

Currently, quality control (QC) of herbal injections based on chemical approach is widely adopted. However, herbal injections are very complex mixtures that contain numerous compounds from multiple different chemical classes, including saponins, alkaloids, and flavonoids (Chau et al., 2009). Thus, based on current chromatographic and mass spectrographic technique, it is very difficult to identify all the compounds of herbal injections (Hao et al., 2008). In addition, because the correlation between chemical information provided by chemical approach and overall *in vivo* activity has not been scientifically justified (Yan et al., 2014), the chemical information lacks enough evidence to ensure clinical safety and efficacy of herbal injections (Fu et al., 2011). Therefore, chemical approach has some defects to control the quality of herbal injections.

Recently, using bioassays to control the quality of complex herbal medicines has been paid attentions. Traditionally, bioassays are extensively and preferably adopted to assure the quality of biological products due to the complexity of their chemical components. Although bioassays are not helpful in chemical information discovery, they have the advantage of offering direct link to bioactivities closely related to clinical safety and efficacy of biological products (Sathish et al., 2013). Nowadays, bioassays have been widely accepted as appropriate methods for QC of biological products by U.S. Pharmacopeia, British Pharmacopoeia, and European Pharmacopeia. Inspired by these facts, we believe that introducing bioassays into the QC system of herbal injections is beneficial to improve their quality standard.

Xueshuantong Lyophilized Powder (XST), consisting of a series of saponins extracted from *Panax notoginseng*, is an herbal injection approved by CFDA (China drug approval number: Z 20025652) and widely applied to treat acute cerebral infarction, stroke, and coronary heart disease in clinic. It can promote blood circulation (Liu et al., 2014), prevent thrombosis (Gui et al., 2013) and dilate blood vessels. The average annual sales of XST in China are about 100 million dollars. However, most adverse reactions including fever, local oozing blood, allergic purpura, and anaphylactic shock (Wang et al., 2016) in clinic are caused by quality problems of XST and have got attention from CFDA (State Food and Drug Administration, 2016). Currently, the main quality standard of XST are general rules of injection and content determination of partial notoginsenosides (ginsenoside Rg1, ginsenoside Rb1, notoginsenoside R1, etc.) which are insufficient to ensure XST quality.

It is well known that the incidence of ADR in clinic is related to several factors including intra-individual variable, drug delivery manner, drug compatibility, and substandard quality. And our study focuses on the quality fluctuation of XST. In this study, an available approach for QC of XST was established by combining chemical analysis with bioassay including antithrombin and proplasmin assay. Ultra-performance liquid chromatography coupled with an electrospray ionization quadrupole time-of-flight mass spectrometry operating in MS^E^ mode (UPLC-QTOF/MS^E^) coupled with data processed by Progenesis QI software, a sensitive, fast, and effective characterization technique of complex systematic components, was used for exploring the potential markers of the different XST, including certainly abnormal, possibly abnormal, and normal XST. The detailed sample information is shown in Table S1.

**Figure 1:**
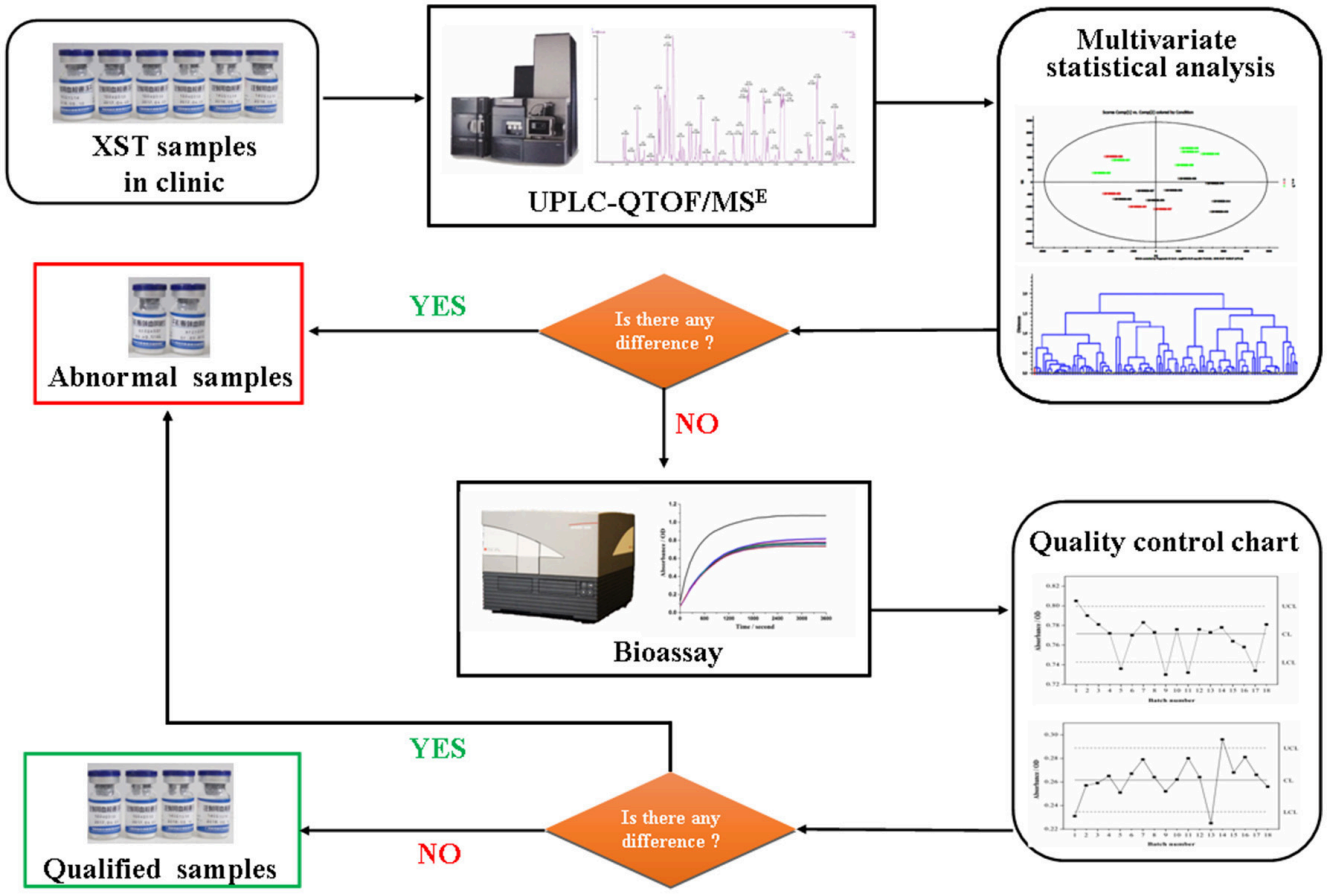
Flowchart for the detection of quality fluctuation of Xueshuantong lyophilized powder (XST) in clinic based on combination chemical approach with bioassay.

Furthermore, according to the therapeutic effects on cardiocerebrovascular diseases of XST and its ADR like local oozing blood, allergic purpura, skin itch, and erythra, bioassays including antithrombin assay and proplasmin assay were carried out to evaluate the differences in bioactivity of the above XST. Integrated analysis of the data from UPLC-QTOF/MS^E^ and bioassay were carried out by using Ezinfo 3.0 and SPSS 20.0 statistics software. The strategy for developing the quality standard of XST is shown in Figure 1.

## Materials and methods

### Chemicals, reagents, and materials

A total of 5 saponins (purity > 98.0%) as reference standards, including notoginsenosides R1 (110745-201518), ginsenosides Rg1 (110703-201529), ginsenosides Re (110754-201525), ginsenosides Rb1 (110704-201522), and ginsenosides Rd (111818-201501) were purchased from National Institutes for Food and Drug Control. HPLC grade acetonitrile and SupraPur grade formic acid were purchased from Thermo Fisher Scientific (Waltham, MA, USA). Ultrapure water (18.2 MΩ cm at 25°C) was prepared by a Milli-Q water purification system (Millipore, Bedford, MA, USA). Leucine-enkephalin, tris-hydroxymethyl-aminomethane-hydrochloric acid (Tris-HCl), fibrinogen, thrombin, and plasmin were purchased from Sigma-Aldrich (St. Louis, MO, USA).

### Preparation of samples

To reflect the clinical reality, we have collected the 18 batches of XST samples (China drug approval number: Z 20025652) from clinic. That is, all the samples were in accord with the current national standard of XST, and then were used in clinic. However, some XST samples had induced ADR due to the quality problem. According to the international ADR causality assessment methods (Karch and Lasagna, 1977; Naranjo et al., 1981), the above samples were divided into three groups: (1) C group, six batches of certainly abnormal XST (inducing ADR in clinic due to their quality problems), numbered with 3, 4, 11, 12, 13, and 17; (2) P group, 4 batches of possibly abnormal XST (inducing ADR in clinic, but their quality problems are uncertainty), numbered with 1, 5, 9, and 14; (3) N group, eight batches of normal XST (no ADR in clinic), numbered with 2, 6, 7, 8, 10, 15, 16, and 18. The clinical information for XST samples with ADR was given in Table S1.

The stock solution was prepared by dissolving the XST sample in ultrapure water (100 mg/mL) and was stored at 4°C prior to analysis. Fibrinogen was dissolved in Tris-HCl (37°C) to prepare the fibrinogen solution (0.5%). Thrombin and plasmin were respectively dissolved in normal saline (37°C) to prepare the thrombin solution (1 U/mL) and plasmin solution (100 U/mL). The fibrinogen, thrombin, and plasmin solutions were freshly prepared and kept at 37°C for the entire experiments.

### UPLC-QTOF/MS^E^ analysis

Analysis was performed on a Waters ACQUITY UPLC I-Class system coupled to a Waters SYNAPT G2-S HDMS QTOF mass spectrometer equipped with an electrospray ionization (ESI) interface (Wang et al., 2015). All the 18 batches of sample solutions (2 μL) were injected into a Waters ACQUITY UPLC BEH C18 column (1.7 μm, 2.1 mm × 100 mm), respectively. The flow rate was set at 0.4 mL/min. The temperature in the auto sampler and in the column oven was set at 10°C and 40°C, respectively. The mobile phase consisted of A (water: formic acid = 100: 0.1, *v*/*v*) and B (acetonitrile: formic acid = 100: 0.1, *v*/*v*), with the following gradient: 0–4.0 min, 90%−75% A; 4.0–3.0 min, 75%−68% A; 13.0–15.0 min, 68%−50% A; 15.0–16.0 min, 50%−0% A; 16.0–17.0 min, 0% A; 17.0–17.1 min, 0%−90% A and 17.1–20.0 min, 90% A.

The high-accuracy MS data were collected from *m/z* 100–1500 Da in both positive and negative MS^E^ continuum modes. The electrospray ionization conditions were set as follows: capillary voltage, 1.0 kV; cone voltage, 40 V; cone gas flow, 50 L/h; source temperature, 120°C; desolvation gas flow, 800 L/h; and desolvation temperature, 500°C. Nitrogen and argon were used as cone and collision gases, respectively. In the case of low energy, collision energy was set at 4 V; in the case of high energy, a collision energy ramp 40–80 V for negative mode and 20–40 V for positive mode were used. The ion acquisition rate was 0.2 s with resolution more than 30,000 full width at half maximum. To ensure the mass accuracy and reproducibility of the optimized MS condition, leucine encephalin (*m*/*z* 554.2615 in negative mode and *m*/*z* 556.2771 in positive mode) was used for lock mass at a concentration of 100 pg/mL and a flow rate of 10 μL/min. The leucine encephalin was sprayed into the MS instrument every 10 s. The instrument was calibrated using sodium formate solution as the calibration standard as suggested by the manufacturer. Data acquisition and processing were carried out with Masslynx™ v4.1 (Waters Co.) and UNIFI® Scientific Information System v1.7 (Waters Co.), respectively.

### Antithrombin assay

XST sample solution (100 mg/mL, 50 μL) was added to the wells in a 96-well microplate, along with 100 μL of fibrinogen solution, and then incubated for 10 min. Normal saline and 10 mmol/L of benzamidine hydrochloride were used as negative and positive controls, respectively. To initiate the polymerization of fibrinogen, 50 μL of thrombin solution (1 U/mL) was added, and then the absorbance of the reaction system was recorded continuously over 60 min on a Spark 10M microplate reader (Tecan, Switzerland) at 660 nm and 37°C.

### Proplasmin assay

Firstly, 100 μL of fibrinogen solution were added to in a 96-well microplate, along with 50 μL of thrombin solution (1 U/mL). The simulative blood coagulation reaction was initiated with the polymerization of fibrinogen for 2 min. And 50 μL of plasmin solution (1 U/mL) and 50 μL XST solution (100 mg/mL) were added successively to indicate fibrinolysis reaction. Then the 96-well microplate was loaded into the microplate reader, which recorded dynamically the absorbance of the fibrinolysis system over 30 min at 660 nm and 37°C. The absorbance values at the end of the reaction were used to reflect the proplasmin activity of the different XST samples.

### Statistical analysis

Partial least squares discriminant analysis (PLS-DA) of UPLC-QTOF/MS^E^ raw data was completed by Ezinfo 3.0 (Waters, Milford, MA, USA). The absorbance values in bioassay including antithrombin assay and proplasmin assay were loaded on SPSS 20.0 statistics software (SPSS Inc., Chicago, IL, USA) to obtain the quality control chart.

## Results

### UPLC-QTOF/MS^E^ analysis

The MS^E^ data of XST samples in negative mode was chosen to be processed by Progenesis QI, because more comprehensive compound ions in XST samples could be detected in negative ionization, which had been proved to have higher sensitivity than positive mode (Wang et al., 2015). Firstly, raw UPLC-QTOF/MS^E^ data for 18 batches of XST samples acquired in both low and high energy were imported into the Progenesis QI software and converted into a 2D ion intensity map with *m/z* as abscissa and retention time as ordinate (Figures S1A,B). Peak intensity and retention time-exact mass pair (RT-EM) were provided respectively as observed values and variables in the 2D matrix. Furthermore, peak alignment was performed in automatic manner setting a sample with common features as the alignment reference. The alignment vector was shown in Figure S1C. The alignment quality evaluation results indicated that the score of all samples were greater than 98%, as shown in Figure S1D. Next, for peak picking, the parameters of “sensitivity,” “minimum peak width,” and “retention time limit” were set as 3, 0.15 min, and default value, respectively, to obtain the maximum real feature ion signals with random noise excluded. As a result, 2,920 peaks (compound ions) were observed including various adduct ions, and for review deconvolution, the adduct ions of same compound were grouped together to improve the accuracy and reliability of result (Figures S1E,F). Finally, the filtered compound ions were exported into EZinfo for further analysis.

**Figure 2:**
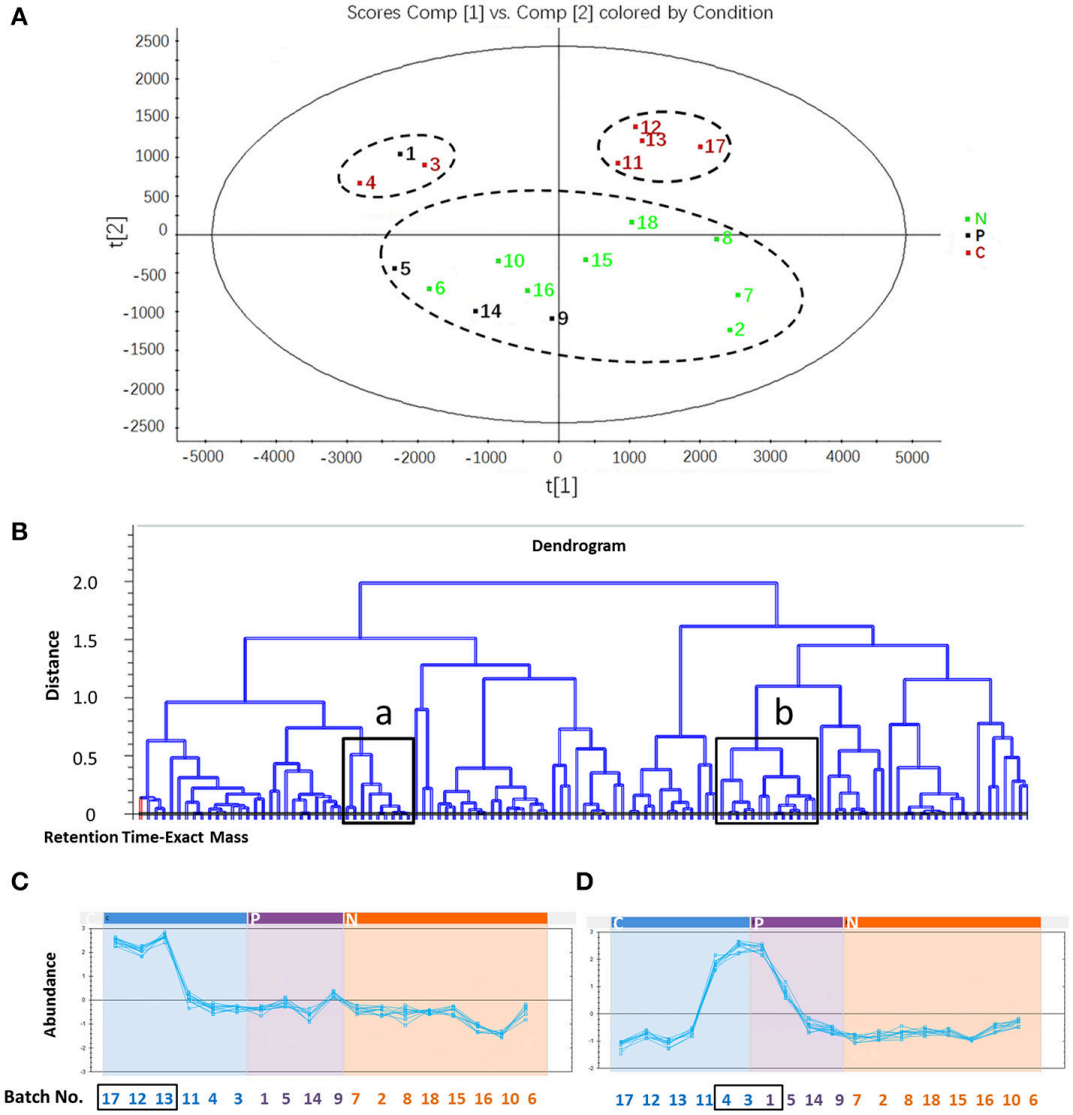
The screening process of potential compounds related to adverse drug reaction. **(A)** Partial least squares-discriminant analysis of samples in C, P and N group. All samples in C group were clustered separately from those in N group. C group, six batches of certainly abnormal XST (inducing ADR in clinic due to their quality problems); P group, four batches of possibly abnormal XST (inducing ADR in clinic, but their quality problems are uncertainty); N group, eight batches of normal XST (no ADR in clinic). **(B)** Dendrogram of 117 identified compounds. “a” and “b” indicate the selected region in which compound abundance would be determined in different sample batches. **(C)** Abundance of compounds in region a of 18 batches of samples. The compound abundance in region a of sample No. 17, 12, and 13 is higher than that in normal samples. **(D)** Abundance of compounds in region b of 18 batches of samples. The compound abundance in region b of sample No. 4, 3, and 1 is higher than that in normal sample.

To find the components responsible for differentiation among C, P, and N groups of XST samples, PLS-DA was applied to develop a discriminant model. Figure 2A and Figure S1G (the score plot) showed that the samples in C group (marked in red) were located separately from the normal samples (N group, marked in green), indicating that there were different chemical compositions in the two groups. In addition, the C group was also divided into two subgroups that were far apart, indicating that the potential chemical markers were different in the two subgroups, although they could all induce ADR due to quality problems. In the loading plot (Figure S1H), each point represented one compound ion (RT-EM), and the distance of the ion point to the origin indicated the relative contribution degree to PLS-DA model. The contribution is expressed as variable importance in projection value (VIP), and compound ions with larger VIP value is considered as more major components accountable for differentiating three groups. As shown in Figure S1H, a total of 117 compound ions with VIP > 1 were screened (marked with red square).

The RT-EM data of 117 screened compound ions were reimported into Progenesis QI for correlation analysis, described as the dendrogram in Figure 2B and Figure S1I, which was created by cluster analysis method in terms of abundance of the differentiated compound ions. The roots represented compound ions, some of which marked with black boxes (region a and b in Figure 2B) were in high content in samples certainly causing ADR but low in normal samples. These compound ions were accounted as potential components responsible for clinical ADR of XST samples (some representative data shown in Figure S1J). The analysis showed that the compound ions in both “region a” and “region b” were considered as potential chemical makers, and their RT-EM data were as follows: (1) region a: 4.01–1094.5901 n, 6.29–1124.5997 n, 6.54–1123.5927 *m/z*, 6.55–1169.5979 *m/z*, 9.22–944.4572 n, 10.09–770.4830 n, 12.51–1107.5986 *m/z*, 15.76–751.4673 *m/z*, and 16.07–784.4948 n; (2) region b: 4.08–1108.6057 n, 4.10–961.5362 *m/z*, 4.12–931.5199 *m/z*, 4.12–932.5386 n, 4.54–961.5364 *m/z*, 5.70–845.4840 *m/z*, 5.70–800.4949 n, 7.32–637.9845 *m/z*, 9.17–1371.6885 *m/z*, 11.15–1239.6406 *m/z*, 11.39–1092.6109 n, 14.16–1077.5890 *m/z*, and 16.22–750.7812 n. And then, these results were carried out to identify the abnormal samples. The No. 17, 12, and 13 in C group could be differentiated by the potential chemical makers in “region a” (Figure 2C), and the No. 4, 3 in C group and No. 1 in P group could be differentiated by the potential chemical makers in “region b” (Figure 2D). Therefore, based on the developed UPLC-QTOF/MS^E^, 83.3% (5/6) certainly abnormal samples inducing ADR in clinic (not all) were successfully identified from the N group, P group and other certainly abnormal samples, suggesting the requirement for assistance of other detection methods. In addition, it could be speculated that No. 1 in P group induced ADR due to its quality problems.

### Structure analysis of potential components related to ADR

The full MS scan provided protonated or deprotonated molecules in their intact form, while the target MS/MS scan provided fragment information. The fragmentation patterns of the reference saponins were investigated first, and the types of aglycone, sequences, and linkage positions of saccharide chains could be deduced accurately according to some diagnostic fragments pathways in negative mode. The main components of XST are panaxnotoginseng saponins (PNS), which mainly include three types: protopanaxadiol (PPD), protopanaxatriol (PPT), and ocotillol (OCO). Their characteristic MS fragments are PPD *m*/*z* 443, 425, 407; PPT *m*/*z* 441, 423, 405, and OCO *m*/*z* 457, 439, 421. The sugar residues of ginsenosides involve in glucose (Glc), rhamnose (Rha), xylose (Xyl) and so on. In negative mode, saponin molecules are ionized into [M–H]^−^ precursor ion. Collision induced dissociations (CID) of [M–H]^−^ ions provided diagnostic information on the successive elimination of the terminal sugar (neutral loss: 162 Da for glc, 146 Da for rha, and 132 Da for xyl/ara) and the product fragments (*m/z* 459 for PPD aglycone and *m/z* 475 for PPT aglycone). More information in detail was given in Figure S2.

Further, the characterizations of potential components (namely, potential chemical markers) related to ADR were following the strategy: unequivocal confirmation was conducted by comparison with the retention time and the fragmentation behavior of reference standards, while tentative identification was speculated by matching with the literature and the SciFinder database. As a result, 14 potential components including ginsenoside Rg1, Rg3, Rb1, and notoginsenoside R1 in both region a and region b were successfully confirmed or tentatively identified. More structural information in detail was given in Table 1 and Figures S3, S4.

**Table 1:** The identification of potential adverse reaction inducers in Xueshuantong lyophilized powder (XST) samples.

**Region**	**No**.	**Compound (RT-EM)**	**Ion species**	**Mass error (ppm)**	**Fragment ions**	**Identification**	**Formula**	**References**
a^*^	1	4.01-1094.5901n	/	2.1	1139.3147 [M+HCOO]^−^, 1093.6591 [M–H]^−^, 931.4712 [M–H–Glc]^−−^**^^, 769.1474 [M–H–Glc–Glc]^−^	Gypenoside LXIX/LXXI/floralginsenoside P	C_53_H_90_O_23_	Li et al., 2015
	2	6.54-1123.5927m/z 6.55-1169.5979m/z 6.29-1124.5997n	[M–H]^−^ [M+FA–H]^−^ /	1.9 1.5 1.2	1123.5927 [M–H]^−^, 961.2576 [M–H–Glc]^−^, 799.4127 [M–H–Glc–Glc]^−^, 781.3677 [M–H–Glc–Glc–H_2_O]	Notoginsenoside A	C_54_H_92_O_24_	Li et al., 2015
	3	10.09-770.4830n	/	0.1	815.1427 [M+HCOO]^−^, 769.3478 [M–H]^−^, 637.3215 [M–H–Xyl/Ara]^−−^***^^, 475.3674 [M–H–Xyl/Ara–Glc]^−^, 391.2145 [M–H–Xyl/Ara–Glc–C_6_H_12_]^−^	Notoginsenoside R2	C_41_H_70_O_13_	Wang et al., 2015
	4	12.51-1107.5986m/z	[M–H]^−^	2.6	1107.5986 [M–H]^−^, 945.1476 [M–H–Glc]^−^, 783.3257 [M–H–Glc–Glc]^−^, 621.3694 [M–H–Glc–Glc–Glc]^−^, 459.3748 [M–H–Glc–Glc–Glc–Glc]^−^	Ginsenoside Rb1	C_54_H_92_O_23_	Chang et al., 2017
	5	15.76-751.4673*m/z*	[M–H]^−^	4.7	797.2149 [M+HCOO]^−^, 751.4673 [M–H]^−^, 619.3610 [M–H–Xyl]^−^, 457.0247 [M–H–Xyl–Glc]^−^	Notoginsenoside T5	C_41_H_68_O_12_	Shen et al., 2016
	6	16.07-784.4948n	/	−3.8	829.3217 [M+HCOO]^−^, 783.7489 [M–H]^−^, 621.0744 [M–H–Glc]^−^, 459.4174 [M–H–Glc–Glc]^−^, 375.6423 [M–H–Glc–Glc–C_6_H_12_] ^−^	Ginsenoside Rg3	C_42_H_72_O_13_	Shen et al., 2016
b^*^	1	4.08-1108.6057n	/	2.0	1107.3674 [M–H]^−^, 945.1247 [M–H–Glc]^−^, 783.6941 [M–H–Glc–Glc]^−^, 765.3201 [M–H–Glc–Glc–H_2_O]^−^	Yesanchinoside E	C_54_H_92_O_23_	Zou et al., 2002
	2	4.10-961.5362m/z 4.54-961.5364m/z	[M–H]^−^ [M–H]^−^	−1.7 −1.5	799.3674 [M–H–Glc]^−^, 781.3270 [M–H–Glc–H_2_O]^−^, 637.3021 [M–H–Glc–Glc]^−^, 475.0107 [M–H–Glc–Glc–Glc]^−^, 391.0369 [M–H–Glc–Glc–Glc–C_6_H_12_] ^−^	Notoginsenoside R3/R6	C_48_H_82_O_19_	Wang et al., 2015
	3	4.12-931.5199m/z 4.12-932.5386n	[M–H]^−^ /	−7.8 3.9	931.5199 [M–H]^−^, 799.3204 [M–H–Xyl]^−^, 637.0236 [M–H–Xyl–Glc]^−^, 475.3207 [M–H–Xyl–Glc–Glc]^−^, 391.3690 [M–H–Xyl–Glc–Glc–C_6_H_12_] ^−^	Notoginsenoside R1	C_47_H_80_O_18_	Wang et al., 2015
	4	5.70-845.4840m/z 5.70-800.4949n	[M+FA–H]^−^ /	−7.5 2.6	845.4840 [M+HCOO]^−^, 799.7456 [M–H]^−^, 637.0214 [M–H–Glc]^−^, 619.0357 [M–H–Glc–H_2_O]^−^, 475.0555 [M–H–Glc–Glc]^−^, 391.0369 [M–H–Glc–Glc–C_6_H_12_]^−^	Ginsenoside Rg1	C_42_H_72_O_14_	Wang et al., 2015
	5	9.17-1371.6885m*/*z	[M–H]^−^	6.1	1371.6885 [M–H]^−^, 1239.0149 [M–H–Xyl]^−^, 1077.0748 [M–H–Xyl–Glc]^−^, 945.3741 [M–H–Xyl–Glc–Xyl]^−^, 783.3668 [M–H–Xyl–Glc–Xyl–Glc]^−^	Notoginsenoside D	C_64_H_108_O_31_	Wang et al., 2015
	6	11.15-1239.6406m*/z*	[M–H]^−^	2.2	1239.6406 [M–H]^−^, 1107.1436 [M–H–Xyl]^−^, 1077.0485 [M–H–Glc]^−^, 945.3204 [M–H–Xyl–Glc]^−^, 783.3267 [M–H–Xyl–Glc–Glc]^−^, 621.2107 [M–H–Xyl–Glc–Glc–Glc]^−^, 459.3647 [M–H–Xyl–Glc–Glc–Glc–Glc]^−^	Ginsenoside Ra3/notoginsenoside Fa/R4	C_59_H_100_O_27_	Wang et al., 2015
	7	11.39-1092.6109n	/	0.2	1091.3214 [M–H]^−^, 929.3644 [M–H–Glc]^−^, 767.0236 [M–H–Glc–Glc]^−^, 605.0270 [M–H–Glc–Glc–Glc]^−^	Notoginsenoside I	C_54_H_92_O_22_	Yang et al., 2017
	8	14.16-1077.5890m*/z*	[M–H]^−^	3.6	1077.5890 [M–H]^−^, 945.3674 [M–H– Ara/Xyl]^−^, 915.3140 [M–H–Glc]^−^, 621.0485 [M–H– Ara/Xyl–Glc–Glc]^−^, 459.3647 [M–H– Ara/Xyl–Glc–Glc–Glc]^−^	Ginsenoside Rb2	C_53_H_90_O_22_	Li et al., 2016

* *Region a and b indicate the selected region in which compound abundance would be determined in different sample batches, as shown in Figure 2B*.

** *Glc, Glucose*.

*** *Xyl, Xylose; Ara, Arabinose*.

### Antithrombin assay and proplasmin assay

The above chemical approach (UPLC-QTOF/MS^E^) did not identify all C group and P group of XST samples from N group. Considering that XST is complex mixtures with biological activity, bioassays including antithrombin assay and proplasmin assay would be used to solve this issue.

**Figure 3:**
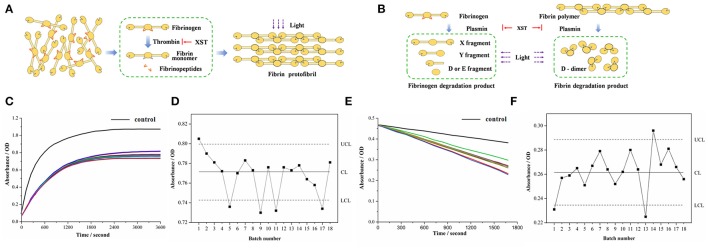
Bioassays based on antithrombin assay and proplasmin assay. **(A)** Principle of antithrombin assay. **(B)** Principle of proplasmin assay. **(C)** Absorbance of each sample based on antithrombin assay. Black curve refers to control group while the other curves reflect absorbance of 18 batches of XST samples. **(D)** Quality control chart reflecting absorbance of each sample based on antithrombin assay. **(E)** Absorbance of each sample based on proplasmin assay. Black curve refers to control group while the other curves reflect absorbance of 18 batches of XST samples. **(F)** Quality control chart reflecting absorbance of each sample based on antithrombin assay.

Thrombin, critical for the blood coagulation system, is a key enzyme of the formation of fibrin clot and a crucial target for various anti-thrombotic studies (Kolodziejczyk et al., 2017). As a proteolytic enzyme, thrombin initiates polymerization of fibrin by cleaving the fibrinopeptides from the physiological substrate (fibrinogen) to obtain fibrin monomer (Janmey et al., 2009). Free from fibrinopeptides, fibrin monomers aggregate and lengthen to produce protofibrils, which further assemble to yield clots (Figure 3A). The above structural modification can alter the light transmittance, and then change over time in the above process. Therefore, it could be monitored by microplate reader to reflect the thrombin activity. In the same way, when XST samples were added respectively, their anti-thrombin activities can be also observed from the alternation of absorbance value.

In addition to blood coagulation system, plasma fibrinolysis system also plays an important role in maintaining circulation of blood. Plasmin can lyse fibrin and fibrinogen in fibrinolysis system. The progressive degradation of fibrinogen by plasmin results in the production of X fragment and Y fragment and generates the terminal products of D fragment and E fragment (Aizawa et al., 2003). By comparison, fibrin is disassembled to D-dimer in Figure 3B. Meanwhile, there is a decrease of absorbance in the above process (Martinez et al., 2013). Thus, it could be applied to assay the proplasmin activities of the different XST samples, and then reflect their quality fluctuation. Therefore, after the application of UPLC-QTOF/MS^E^, the bioassays were performed for identifying the XST samples from C group and P group.

On visual inspection, the curves of coagulation or fibrinolytic system affected by the different XST samples exhibited some distinctions (Figures 3C,E). The difference of absorbance in the end of reactions needed to be further determined whether they were accidental errors or abnormal fluctuations. Therefore, quality control chart (Abujiya and Muttlak, 2004) was applied to evaluate the quality of samples according to statistical analysis of the absorbance. In the quality control chart, CL (Center Line) represents the mean absorbance value of XST samples, while UCL (Upper Control Limit), and LCL (Lower Control Limit) represent “±3σ”, namely three standard deviations of absorbance (Figures 3D,F). Generally, parameters distributing out of the range of 3σ indicate the corresponding situations are instable. In this study, in terms of bioactivity analysis, absorbance value below the LCL indicated the antithrombin and/or proplasmin activity of corresponding sample were stronger than normal one, implying the risk of ADR in clinic. On the other hand, the antithrombin and/or proplasmin activity of sample would be weaker if corresponding absorbance value was beyond the UCL, demonstrating that risk of instable clinical effectiveness exists thanks to the low bioactivity. Generally, the absorbance beyond or below the “3σ” range indicates that there are quality problems in the XST samples.

In Figure 3D, the No. 11 and 17 in C group were successfully identified with quality problem, which was consistent with clinical information of XST samples. The two batches samples inducing ADR in clinic due to their quality problem were lower than the LCL in absorbance, indicating these samples had excessive antithrombin activities. That is to say, when the antithrombin activity of XST samples is excessive, they can also cause the clinical adverse reactions (such as capillary hemorrhage, erythema). Interestingly, the No. 1, 5, and 9 in P group could be differentiated by antithrombin assay, and then it could be speculated that the three samples in P group induced ADR due to their quality problems.

Similarly, in Figure 3F, the No. 13 in C group was identified again with quality problem. As we know, the sample inducing ADR in clinic due to its quality problem was lower than the LCL in absorbance. Namely, when the proplasmin activity of XST samples is excessive, they can also cause the above adverse reactions. Furthermore, the No. 1 and 14 in P group could also be identified by proplasmin assay, and it suggested that owing to quality problem, the two samples in P group could induce clinical ADR. In addition, the results of all the samples in N group were within the normal range (“±3σ”) based on antithrombin assay and proplasmin assay. Therefore, based on the more efficient bioassays including antithrombin assay and proplasmin assay, it suggested that all the possibly abnormal samples were closely related to their quality fluctuation, and then successfully identified from the N group, C group and other P group.

### Result integration

**Figure 4:**
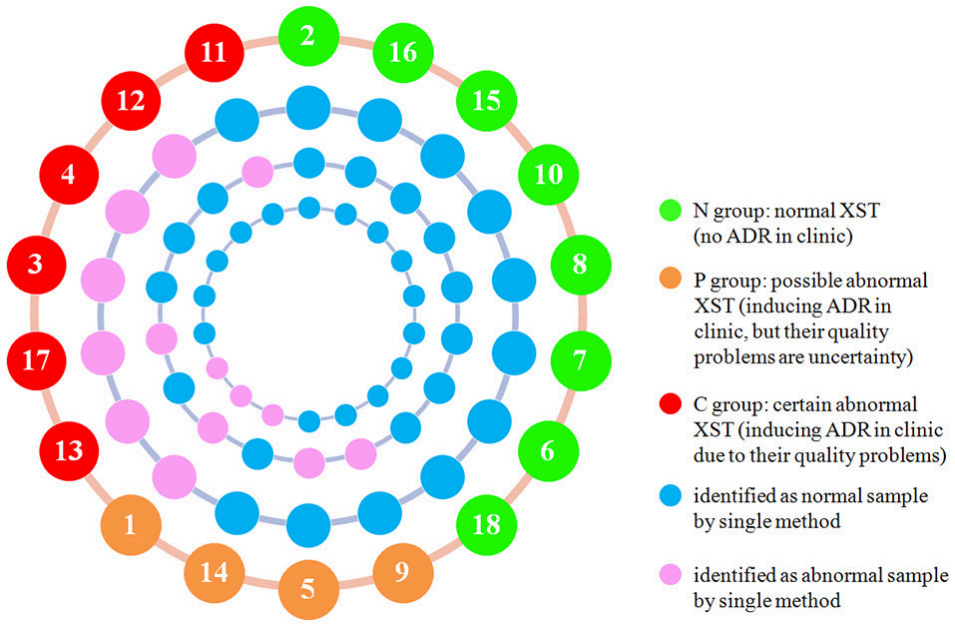
Representation of integration results of UPLC-QTOF/MS^E^, antithrombin assay, and proplasmin assay. From inside to outside, the circles show the proplasmin assay results, antithrombin assay results, UPLC-QTOF/MS^E^ results, and sample information (Sample number and group as indicated by the colors in the key).

Although all the above methods divided the samples into various groups, no single method could differentiate all the C group and P group from the N group. For a sample that was classified as abnormal sample by any detection method, it would finally be classified as abnormal sample no matter how many other methods classify it as normal sample. By adopting this method, all the XST samples in C group and P group were identified successfully from the N samples (Figure 4), which suggests that this integration method is appropriate for integrating the results of the UPLC-QTOF/MS^E^ and bioassays including antithrombin assay and proplasmin assay.

## Discussion

Currently, chemical approaches are mainly adopted used in QC of herbal injections such as XST. Due to their complex chemical mixture, it is sometimes difficult to identify all the components of herbal injections using chemical approaches (Hao et al., 2008). In the study, we combined bioassays with UPLC-QTOF/MS^E^ for QC of XST and found out that although no single method could differentiate all the samples in C group and P group, combination of different detection methods could discriminate all the above samples. Therefore, the bioassays are necessary and useful supplement to develop the QC of XST.

As we know, XST are complex mixtures containing with many components. However, the relationships between their components and clinical efficacy/ADR remain unclear. In our study, optimized conditions of mass spectrum and 14 potential components responsible for clinical ADR were successfully established and identified by UPLC-QTOF/MS^E^, respectively. Most of the samples in C group (No. 3, 4, 12, 13, and 17) and No. 1 in P group had different total ion chromatograms with some more intense peaks than those observed in N group. The compounds included in these peaks were identified as ginsenoside Rg1, Rg3, Rb1, and notoginsenoside R1 which were major saponins in XST, suggesting these compounds could be responsible for the ADR of XST in clinic. More structural information in detail was given in Table 1. Interestingly, ginsenoside Rg1, Rg3, Rb1, and notoginsenoside R1 are known to inhibit the activities of thrombin, thrombosis, and platelet aggregation (Dong et al., 2005; Lee et al., 2008; Zhou et al., 2014). All these activities are closely related to curative effects of XST. However, on the other hand, when the contents of these components are excessive, they can also cause side effects (such as subcutaneous hemorrhage, erythema). This phenomenon contrasts with our commonly hold view that the higher the active component content is, the higher the quality is. The reason might be that the contents of these components have exceeded the maximal safe dose in XST. Besides, in Chinese Pharmacopoeia, most herbal medicines only have minimum limit. Therefore, our finding would be helpful for our understanding of the importance of content to ADR and for us to set proper quality standards of herbal medicines.

Promoting blood circulation is the major pharmacological effect of XST. Thrombin and fibrinogen play important roles in hemostasis, and their disorders can result in life-threatening bleeding and coronary and cerebral thrombosis. Fibrin polymerization is necessary for hemostasis and involves conversion of fibrinogen into fibrin to form aggregated fibers under thrombin catalysis (Chernysh et al., 2012). To further discriminate the ADR samples, we then evaluated the interactions between thrombin/proplasmin and XST, respectively. Interestingly, unlike N group, all the samples in P group (No. 1, 5, 9, and 14) and half of the samples in C group (No. 11, 13, and 17) identified by the bioassays exhibited abnormal bioactivities. The results suggested that the bioactive characteristics of these samples were not consistent with the detected chemical information. For example, No. 11 in C group, which was consistent with N group according to the UPLC-QTOF/MS^E^, wondrously exhibited different bioactive to N group. As far as we know, a high dosage (or strength) of the anticoagulant such as thrombin inhibitor is closely related to the ADR (e.g., subcutaneous hemorrhage and erythema; Crowther and Warkentin, 2008; Baber et al., 2015). Considering the symptoms of ADR samples in clinic (Table S1), we believe that abnormal bioactivity is correlated with the ADR of XST. Therefore, the bioassays including antithrombin assay and proplasmin assay are suitable method for developing the QC of XST. This means that the bioassay should be mainly applied to herbal injections containing complex components that are difficult to analyze using conventional chemical approaches.

Figure 4 showed that combination of the bioassays and UPLC-QTOF/MS^E^ could identify more ADR samples due to quality problems (100% identified samples in both C group and P group) than UPLC-QTOF/MS^E^ alone (60% identified samples in both C group and P group). Even if bioassay is based on *in vitro* studies and cannot include all biological activities of XST, bioassay can represent the primary biological activity, and reflect the available information on the safety and efficacy in clinic. Therefore, we believe that it is of practical significance to integrate bioassays into the conventional QC system of XST.

In our study, two facts should also be noted. The first fact has to do with the number of components identified, especially potential chemical marker related to bioactivity. Totally 14 potential components responsible for clinical ADR of XST were identified in this study, and we believe more components and abnormal samples could be identified by more advanced analytical techniques and their optimized parameters in the future. However, in a sense, it would be an endless job to identify “all the compounds” of XST. Because of the complex chemical mixture, the so-called “all the compounds” of XST are unknown. In addition, there would be another problem of practicability for chemical detection that how to use a large number of components to set a proper standard for QC, while the obtaining of a great number of reference substances is still a practical issue. Therefore, we put more emphasis on bioassay to develop the quality standard of XST.

The second fact is that two different methods (UPLC-QTOF/MS^E^ and bioassays) were applied to control the quality of XST, which seems to hamper the practicability of the integrated approach. In fact, QC of herbal injections is a systemic work that contains other aspects such as acidity or alkalinity, related substances and bacterial endotoxins. Besides, as aforementioned, the bioassays including antithrombin assay and proplasmin assay are automated high-throughput methods. Correspondingly, while improving the quality control of XST, they will not greatly increase the detection time or cost. Therefore, the integrated method is practical, and can promote the quality standard of XST.

Besides, we need to point out that the number of samples from clinic is less in this study, and in a sense, the representativeness of the results might be affected. Although more samples collected from more hospitals are required, the study has been believed to be beneficial exploration for improving the QC of herbal injections.

In summary, we applied chemical approach (UPLC-QTOF/MS^E^) and two bioassays (antithrombin assay and proplasmin assay) to identify the different XST including C group, P group, and N group in clinic. We found out that (1) ginsenoside Rg1, Rg3, Rb1, and notoginsenoside R1 were mainly the possible components responsible for clinical ADR; (2) bioassays could identify the quality fluctuation of samples related to ADR which chemical approach could not; (3) most of the abnormal samples could be identified by integration method. Therefore, we believe that it is advisable to integrate chemical approach and bioassay to develop the quality of XST, and it can be a useful reference for evaluating other herbal injections.

## Author contributions

D-LM and DY conceived of and proposed the idea. Z-RY, Z-HW, and J-FT designed the study. Z-RY, Z-HW, YY, W-WF, and Z-YS performed the experiments. Z-RY, Z-HW, X-TM, and DY participated in data analysis. J-FT supplied XST samples. Z-RY, S-JY, CP, and DY contributed to writing, revising and proof-reading the manuscript. All authors read and approved the final manuscript.

### Conflict of interest statement

The authors declare that the research was conducted in the absence of any commercial or financial relationships that could be construed as a potential conflict of interest.
