# Supplementary material for: UPLC-QTOF/MSE and Bioassay Are Available Approaches for Identifying Quality Fluctuation of Xueshuantong Lyophilized Powder in Clinic
Source: Front Pharmacol. 2018 Jun 15;9:633. doi: 10.3389/fphar.2018.00633 (PMC6013573; doi:10.3389/fphar.2018.00633)
Supplement: Supplementary file 1 [file Data_Sheet_1.DOC]

**Supplementary Material**

**UPLC-QTOF/MS^E^ and Bioassay are Available Approaches for Identifying Quality Fluctuation of Xueshuantong Lyophilized Powder in Clinic**

Zhi-Rui Yang^1#^, Zi-Hao Wang^1,2#^, Jin-Fa Tang^3#^, Yan Yan^2^, Shi-Jun Yue^1^, Wu-Wen Feng^4^, Zheng-Yuan Shi^1^, Xin-Tong Meng^1^, Cheng Peng^4^, Chang-Yun Wang^5^, Da-Li Meng^2*^ and Dan Yan^1*^

^1^ Beijing Key Laboratory of Bio-characteristic Profiling for Evaluation of Rational Drug Use, Beijing Shijitan Hospital, Capital Medical University, Beijing 100038, China

^2^ School of Traditional Chinese Materia Medica, Shenyang Pharmaceutical University, Shenyang 110016, China

^3^ Department of Pharmacy, the First Affiliated Hospital of Henan University of Chinese Medicine, Zhengzhou 450000, China

^4^ Department of Pharmacy, Chengdu University of Traditional Chinese Medicine, Chengdu 611137, China

^5^ Key Laboratory of Marine Drugs (Ministry of Education of China), School of Medicine and Pharmacy, Ocean University of China, Qingdao, China

^#^ These authors contribute equally to this article.

^*^ Correspondence: mengdl@163.com (Da-Li Meng), yd277@126.com (Dan Yan)

**
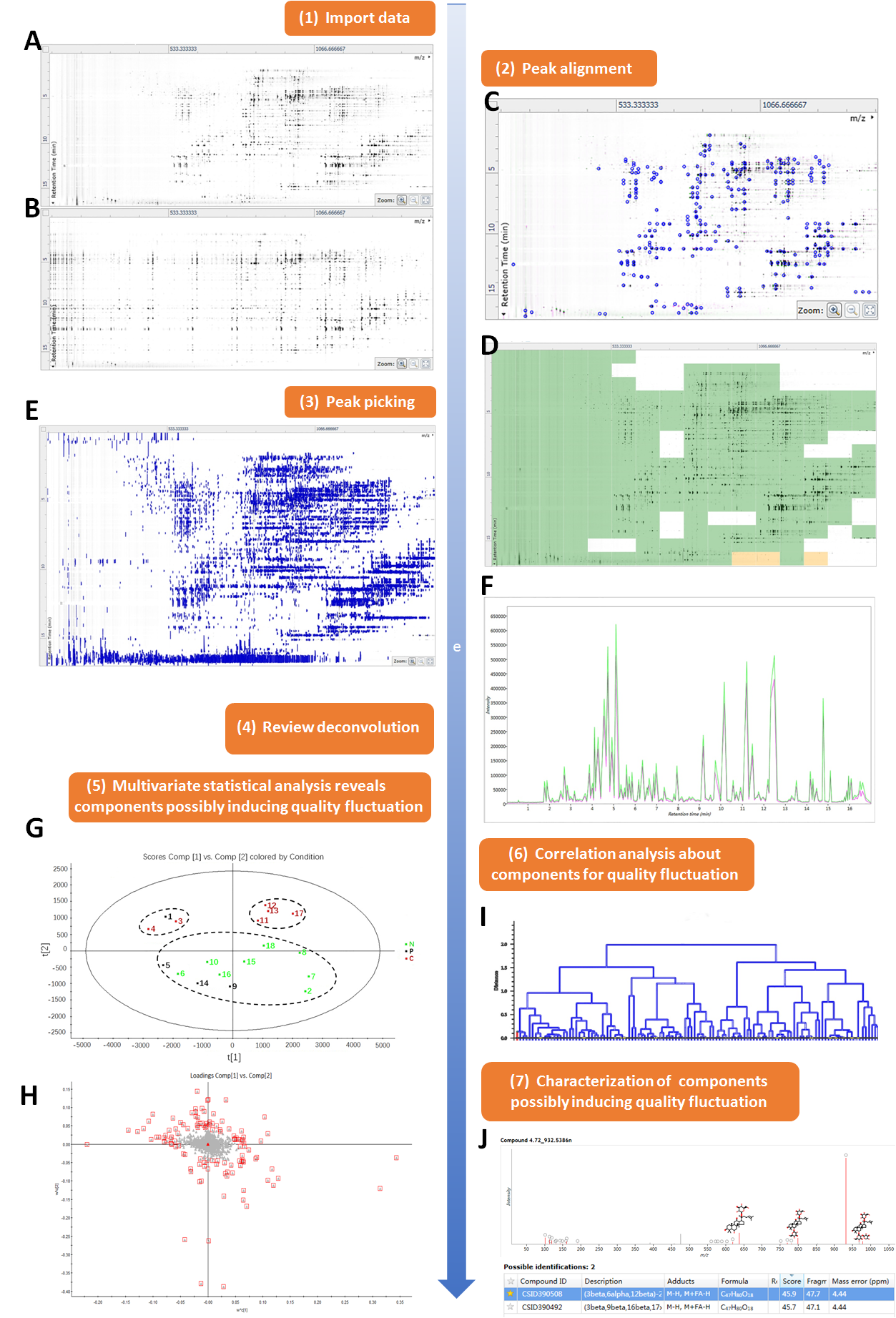
**

**Fig. S1** **|** MS data processing and analysis in UPLC-QTOF/MS^E^. **(A)** data acquired in low collision energy. **(B)** data acquired in high collision energy. **(C)** alignment vector. **(D)** alignment quality evaluation. **(E)** peak picking. **(F)** total ion chromatography of alignment reference and typical batches. **(G)** partial least squares discriminant analysis. **(H)** loading plot. Compound ions with variable importance in projection value (VIP) > 1 were marked in red. **(I)** dendrogram created by cluster analysis. **(J)** characterization of components possibly inducing quality fluctuation.


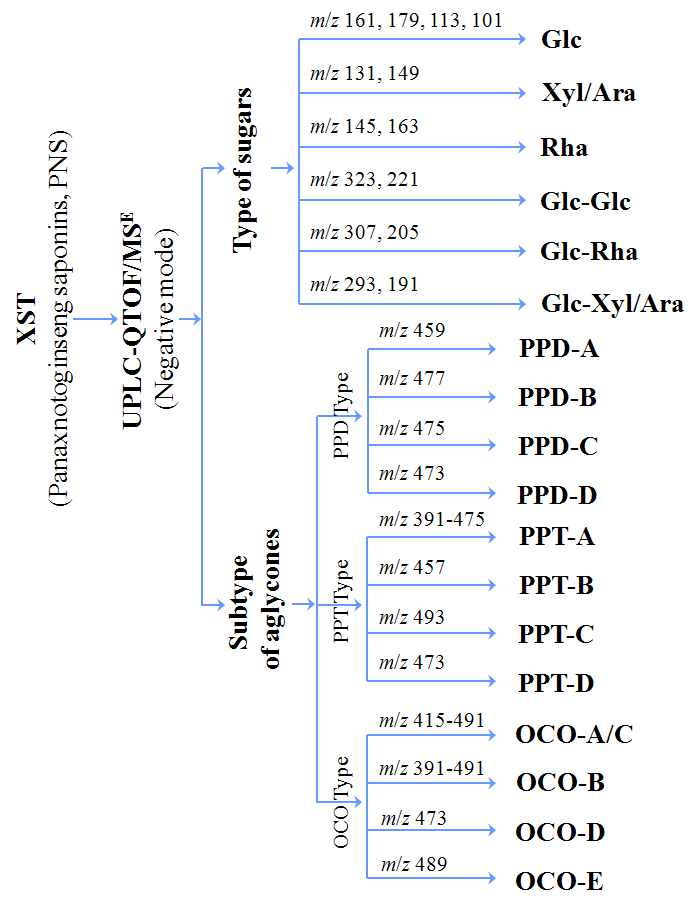


**Fig. S2 |** Characteristic ions of saponins in Xueshuantong lyophilized powder in MS/MS mode. Glc: glucose. Xyl: xylose. Ara: Arabinose. Rha: rhamnose. PPD: protopanaxadiol. PPT: protopanaxatriol. OCO: ocotillol.


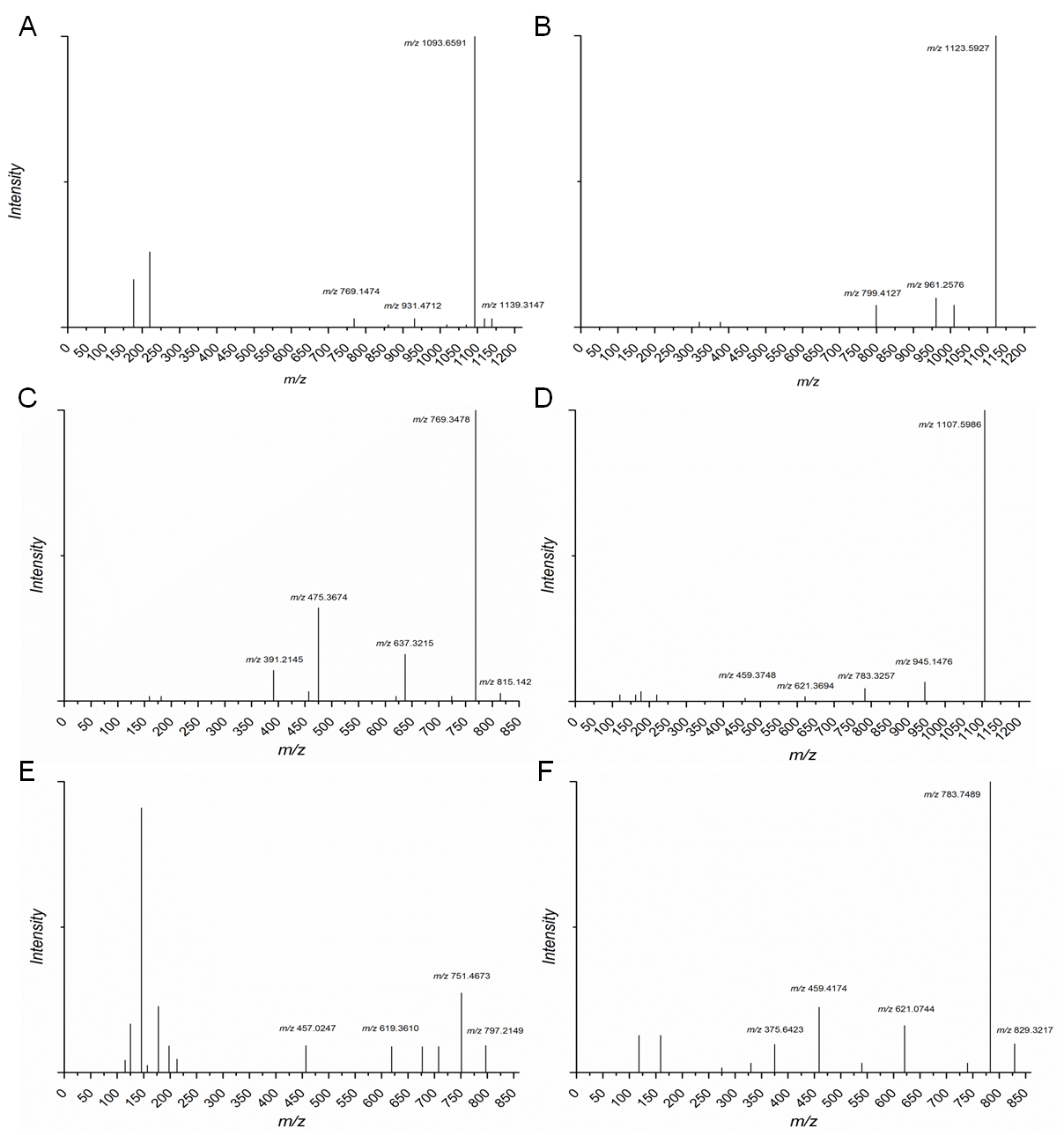


**Fig. S3 |** MS/MS spectra of the potential biomarkers identified in region a. **(A)** gypenoside LXIX/LXXI or floralginsenoside P. **(B)** notoginsenoside A. **(C)** notoginsenoside R2. **(D)** ginsenoside Rb1. **(E)** notoginsenoside T5. **(F)** ginsenoside Rg3.

**
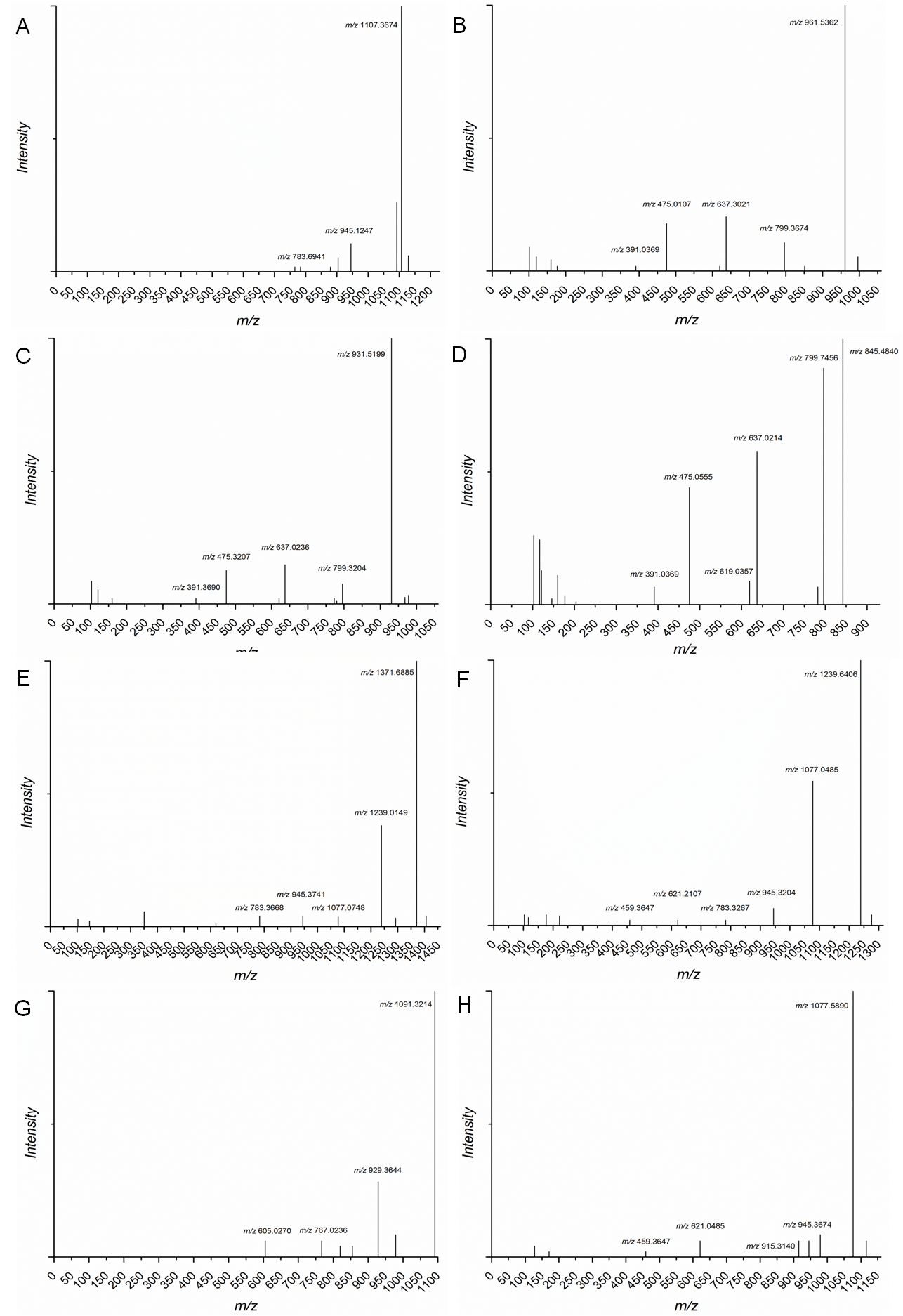
**

**Fig. S4 |** MS/MS spectra of the potential biomarkers identified in region b. **(A)** yesanchinoside E. **(B)** notoginsenoside R3/R6. **(C)** notoginsenoside R1. **(D)** ginsenoside Rg1. **(E)** notoginsenoside D. **(F)** ginsenoside Ra3 or notoginsenoside Fa/R4. **(G)** notoginsenoside I. **(H)** ginsenoside Rb2.

**Table S1** **|** Clinical information for Xueshuantong lyophilized powder (XST) samples with adverse drug reactions (ADR).

| **Patient information** | | | | |  | **Medication information** | | | |
| --- | --- | --- | --- | --- | --- | --- | --- | --- | --- |
| **Hospital  number** | **In-patient  flow number** | **Age** | **Gender** | **Original**  **diseases** |  | **Batch**  **No.** | **Research**  **No.** | **Administration  period (day)** | **Adverse**  **reactions** |
| 1 | 3199 | 64 | male | chronic renal failure and coronary heart disease |  | 15020405 | 1 | 11 | swelling and erythema |
| 2 | 8249 | 47 | female | protrusion of lumbar intervertebral disc |  | 15031214 | 3 | 2 | erythema and pruritus |
| 3 | 24079 | 52 | male | fracture |  | 15031214 | 3 | 1 | erythema and dyspnea |
| 4 | 18545 | 55 | male | recurrent dislocation of knee-cap |  | 14080314 | 4 | 1 | pruritus and rash |
|  | 18552 | 44 | female | cervical spondylosis |  | 14080314 | 4 | 1 | red flake rash |
|  | 18591 | 34 | female | fracture in left tibiofibula |  | 14080314 | 4 | 8 | phlebitis |
|  | 18806 | 31 | male | optic atrophy |  | 14080314 | 4 | 1 | rash |
|  | 18818 | 38 | male | lumbar burst fracture |  | 14080314 | 4 | 10 | maculopapule and erubescence |
|  | 19095 | 63 | female | fracture in right ankle |  | 14080314 | 4 | 12 | erythema and pruritus |
|  | 19106 | 48 | female | medial collateral ligament injury of left knee joint |  | 14080314 | 4 | 14 | rash |
|  | 19211 | 51 | female | right tibial plateau |  | 14080314 | 4 | 8 | rash and pruritus |
| 5 | 21047 | 32 | male | numbness of limb and nephritis |  | 14101315 | 5 | 1 | rash and pruritus |
| 6 | 6818 | 39 | female | protrusion of lumbar intervertebral disc |  | 14090414 | 14 | 8 | flatulence and pruritus |
|  | 6846 | 53 | male | skin soft tissue laceration |  | 14090414 | 14 | 2 | rash |
| 7 | 829 | 63 | female | gouty arthritis |  | 14122404 | 9 | 9 | swelling and pain in elbow joint |
| 8 | 25365 | 62 | male | left clavicle fracture |  | 15040211 | 11 | 7 | rash and pruritus |
|  | 25454 | 29 | female | left patellar dislocation |  | 15040211 | 11 | 5 | pruritus erythe and mamaculopapule |
| 9 | 21793 | 37 | male | dizzy giddy |  | 15050914 | 12 | 1 | headache and capillary hemorrhage |
|  | 21841 | 56 | female | cerebral infarction |  | 15050914 | 12 | 18 | rash and pruritus |
| 10 | 960 | 60 | female | coronary heart disease |  | 15050115 | 13 | 2 | rash |
|  | 985 | 59 | male | coronary heart disease |  | 15050115 | 13 | 4 | fever and erythema |
| 11 | 21329 | 51 | female | coronary heart disease |  | 15050613 | 17 | 2 | chest distress and erythema |
